# Sandwich ELISA for quantitative detection of human collagen prolyl 4-hydroxylase

**DOI:** 10.1186/1475-2859-9-48

**Published:** 2010-06-17

**Authors:** Ekaterina Osmekhina, Antje Neubauer, Katharina Klinzing, Johanna Myllyharju, Peter Neubauer

**Affiliations:** 1 Bioprocess Engineering Laboratory, Department of Process and Environmental Engineering, P.O.Box 4300, FIN-90014 University of Oulu, Finland; 2 Oulu Center for Cell-Matrix Research, Biocenter Oulu and Department of Medical Biochemistry and Molecular Biology, P.O.Box 5000, FIN-90014 University of Oulu, Finland; 3 Laboratory of Bioprocess Engineering, Department of Biotechnology, Technische Universität Berlin, Ackerstr. 71-76, D-13355 Berlin, Germany

## Abstract

**Background:**

We describe a method for specific, quantitative and quick detection of human collagen prolyl 4-hydroxylase (C-P4H), the key enzyme for collagen prolyl-4 hydroxylation, in crude samples based on a sandwich ELISA principle. The method is relevant to active C-P4H level monitoring during recombinant C-P4H and collagen production in different expression systems. The assay proves to be specific for the active C-P4H α_2_β_2 _tetramer due to the use of antibodies against its both subunits. Thus in keeping with the method C-P4H is captured by coupled to an anti-α subunit antibody magnetic beads and an anti-β subunit antibody binds to the PDI/β subunit of the protein. Then the following holoenzyme detection is accomplished by a goat anti-rabbit IgG labeled with alkaline phosphatase which AP catalyzes the reaction of a substrate transformation with fluorescent signal generation.

**Results:**

We applied an experimental design approach for the optimization of the antibody concentrations used in the sandwich ELISA. The assay sensitivity was 0.1 ng of C-P4H. The method was utilized for the analysis of C-P4H accumulation in crude cell extracts of *E. coli *overexpressing C-P4H. The sandwich ELISA signals obtained demonstrated a very good correlation with the detected protein activity levels measured with the standard radioactive assay. The developed assay was applied to optimize C-P4H production in *E. coli *Origami in a system where the C-P4H subunits expression acted under control by different promoters. The experiments performed in a shake flask fed-batch system (EnBase^®^) verified earlier observations that cell density and oxygen supply are critical factors for the use of the inducer anhydrotetracycline and thus for the soluble C-P4H yield.

**Conclusions:**

Here we show an example of sandwich ELISA usage for quantifying multimeric proteins. The method was developed for monitoring the amount of recombinant C-P4H tetramer in crude *E. coli *extracts. Due to the specificity of the antibodies used in the assay against the different C-P4H subunits, the method detects the entire holoenzyme, and the signal is not disturbed by background expression of the separate subunits.

## Background

A sandwich Enzyme-Linked ImmunoSorbent Assay (ELISA) is a powerful tool for quantifying proteins and qualifying their state of activation in complex biological samples. The assay is widely used in clinical diagnostic, food samples analyzing and as a microarray in proteomic applications [1].

The method is based on the detection of hybridization events between two antibodies (capture and detection) and the target proteins. The capture antibodies are used to immobilize the protein onto a solid support and the detection antibodies are recognized by the enzyme-linked secondary antibodies. The linked enzyme catalyzes substrate transformation reactions with generation of a detectable signal.

The sandwich ELISA has certain advantages compared to a standard ELISA: firstly, the ability to use crude or impure samples and still selectively bind an antigen of interest; and, secondly, a better specificity since the antibodies against different epitopes of a target protein are used. We apply the sandwich ELISA for complex proteins measuring in crude cell extracts. In this case the capture and detection antibodies are specific to different subunits of a target protein, due to which only proteins containing both subunits are sensed using the assay.

Here we describe a sandwich ELISA for recombinant human collagen prolyl 4-hydroxylase (C-P4H) detection in crude cell extracts.

Collagen prolyl 4-hydroxylases play a central role in the synthesis of collagens and collagen-like proteins. The human C-P4Hs are α_2_β_2 _tetramers with a total size of 240 kDa. The α subunits contain the catalytic sites and the β subunits keep the protein in a soluble and active state. The β subunit is identical to the enzyme and chaperone protein disulphide isomerase (PDI) [2], one of the most abundant proteins in the endoplasmic reticulum.

Serum C-P4H levels increase in patients with liver cirrhosis, alcoholic hepatitis, acute hepatitis, hepatocellular carcinoma, and cholestatic diseases, and it can be used as a biochemical marker for these diseases [3-6]. As C-P4H is a potential target for treatment of fibrotic diseases, a big interest exists in recombinant expressed C-P4H used for detailed functional and structural studies. Furthermore, C-P4H coexpression is required for recombinant collagen production in different expression systems. An active recombinant human C-P4H tetramer assembly has been successfully achieved in various cell types for above mentioned investigations [7-14]. C-P4H can be efficiently expressed and assembled in yeast, plant and animal cells, but the product yields are rather low. Therefore, recombinant expression systems using the well characterized and fast growing bacterium *Escherichia coli *as a host organism were developed, and they are aimed at large scale production of the target enzyme in high cell density cultivations [14-16].

In such production systems, C-P4H accumulation and activity was monitored by Western blotting, the enzyme activity measurement in radioactive [17] and radioactivity-free [18] assays, analysis of α and β subunits expression at mRNA level with sandwich hybridization [16]. But the exact level of the produced C-P4H tetramer can be accurately measured only after HPLC purification of the protein.

A commercially available immunoassay (Fuji Chemicals, Toyama, Japan; [19]) used for the C-P4H analysis in serum is based on the process of protein capturing and detection via the PDI/β subunit. This method detects both C-P4H tetramers and free PDI/β subunits, but it is entirely unable to distinguish between them. Since PDI/β subunit expression is induced before the α subunit one during recombinant C-P4H production in *E. coli *cells, the PDI/β subunit proves to be synthesized in a large excess over the α subunit [14]. The α subunits aggregate immediately if they are recombinantly produced alone or if the C-P4H tetramer is dissociated [2]. The PDI/β subunit is also present in excess amounts relative to the α subunit *in vivo*.

In the sandwich ELISA described here the capture and detection antibodies are specific to the C-P4H α and PDI/β subunits respectively. The assay is able to detect only proteins containing both subunits and can be performed with unpurified crude cell extracts. The developed method was optimized by using an experimental design approach, evaluated by measuring the levels of recombinant C-P4H produced in *E. coli *cells and applied in optimization of recombinant C-P4H production in *E. coli*.

## Results

### Method development

**Figure 1:**
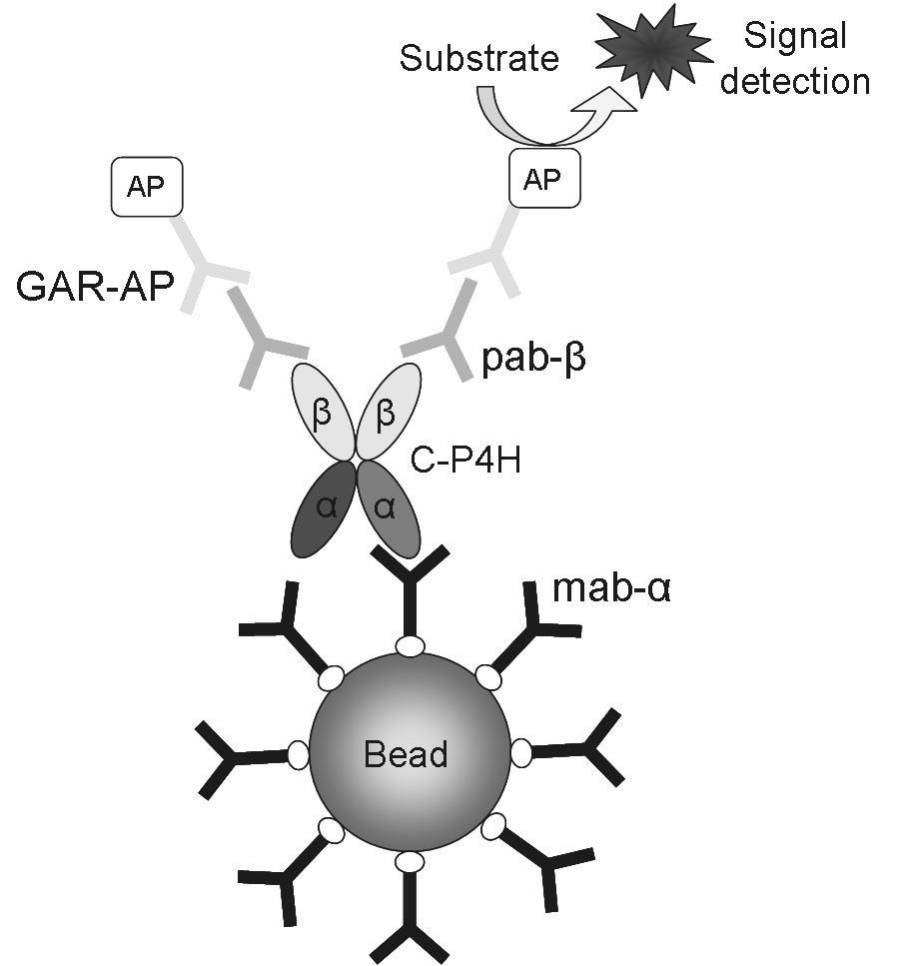
**The principle of bead-based sandwich ELISA for C-P4H**. C-P4H tetramer is captured by the monoclonal antibody against the α subunit (mab-α) coupled to magnetic beads. After a washing step rabbit polyclonal antibody against the β subunit (pab-β) binds to the protein. Goat anti-rabbit IgG labeled with alkaline phosphatase (GAR-AP) is used for the complex detection. The AP catalyzes reactions of substrate transformation with fluorescence signal generation.

The principle of the method is drawn in figure 1. At first the C-P4H binds to capture antibodies coupled with magnetic beads. The monoclonal antibodies against the α subunit (mab-α) were chosen for protein capturing to avoid the system blocking with abundant free PDI/β subunits. The amount of free PDI/β subunits in C-P4H producing *E. coli *cells is high since the production starts with PDI/β subunits expression to keep the later expressed α subunits in a soluble form [14]. After the binding step the complex is washed and incubated with detection antibodies. The protein detection with polyclonal antibody against another C-P4H subunit (PDI/β) guarantees that only proteins containing both α and PDI/β subunits will be measured. Goat Anti-Rabbit IgG labeled with alkaline phosphatase (GAR-AP) attaches to the detection antibody, and the alkaline phosphatase catalyzes substrate transformation reactions with fluorescent products generation. The fluorescence signal is proportional to the C-P4H concentration.

### Mab-α attachment to magnetic beads

**Figure 2:**
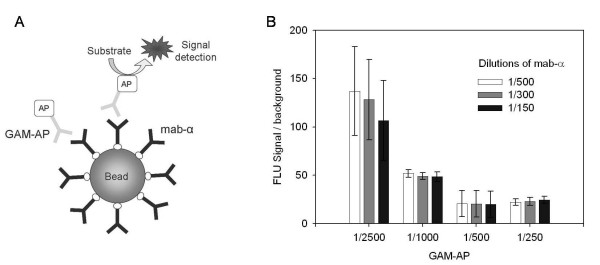
**Attachment of mab-α to magnetic beads and influence of mab-α and GAM-AP dilutions on fluorescent signal**. (A) The principle of the test. The mab-α binds to magnetic beads via biotin-streptavidin interaction. The complex is detected with the Goat Anti-Mouse IgG labeled with AP (GAM-AP). (B) The generated signals obtained when different mab-α and GAM-AP concentrations were used. The error bars show the ± SD of three parallel experiments.

The mab-α labeling with biotin molecules and their attachment onto the streptavidin bead surface were tested (Figure 2A). Therefore streptavidin coated magnetic beads were incubated with different amounts of the biotinylated mab-α. The antibody attached to the beads via the biotin-streptavidin interaction. The formed complex was washed and detected with the Goat Anti-Mouse IgG labeled with alkaline phosphatase (GAM-AP).

Different mab-α and GAM-AP antibodies concentrations were used in the experiment. Generated fluorescent signals proved to be close to the maximum ones ever observed for the bead-based fluorescent assay irrespective of the antibodies dilutions (data not shown). Background fluorescence obtained without adding mab-α was 20-140 times lower compared to specific signals. The best relation between the signal and background (FLU Signal/background ratio) was achieved with the most diluted mab-α (dilution 1/500) and GAM-AP (dilution 1/2500) (Figure 2B) since the background fluorescence grew with increasing of the antibodies concentrations. It was decided to select the exact optimal antibodies concentrations in the sandwich ELISA using the C-P4H tetramer as a target. 1/500 dilution of the mab-α and 1/2500 dilution for the secondary detection antibody were chosen for further experiments.

### Choosing the optimal pab-β

**Figure 3:**
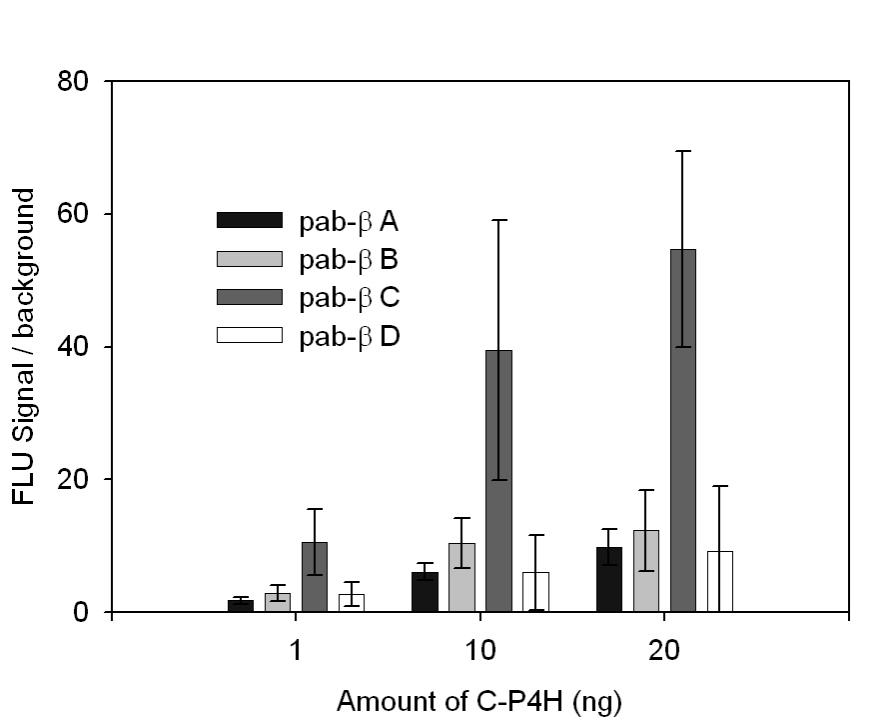
**Comparison of the detection antibodies produced by different manufacturers**. Purified C-P4H was measured with the sandwich ELISA using different pab-β antibodies: pab-β A (Abcam), pab-β B (Calbiochem, Germany), pab-β C (Santa Cruz Biotechnology, Inc.) and pab-β D (Stressgen Bioreagents). The error bars show the ± SD of three parallel experiments.

Four different polyclonal antibodies against human PDI were tested as the C-P4H sandwich ELISA detection antibody (Figure 3). 1, 10 and 20 ng of purified C-P4H were used as a target. The antibodies dilutions were as follows: 1/500 for mab-α and for each pab-β, and 1/5000 for GAR-AP used here as a secondary detection antibody. The secondary antibody (GAR-AP) concentration was chosen lower than for the GAM-AP antibody used in the previous experiment since the less amount of detectable complexes were expected here.

The signal/background ratios were compared for each pab-β. This parameter was about 3 times higher when pab-β C was used; therefore this antibody was selected for further experiments.

A big variability observed for the results was due to clumping of the beads in some experiments. To avoid this effect, increase the efficiency of the washing and the sensitivity of the assay, the method was optimized in the followed experiment.

### Optimization of the method

**Figure 4:**
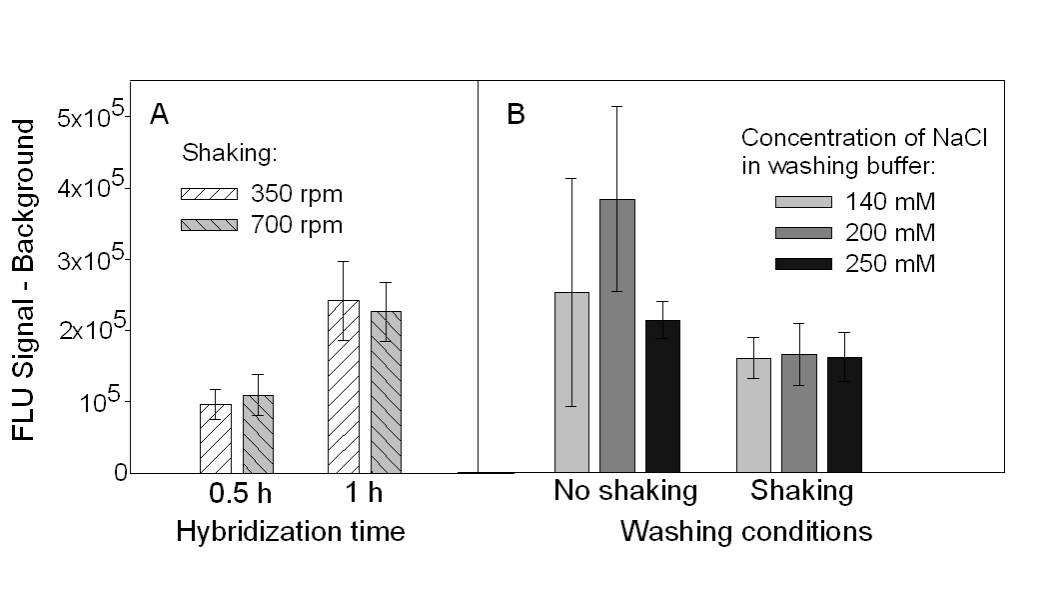
**Optimization of hybridization and washing procedures of the C-P4H sandwich ELISA**. (A) Optimization of the hybridization time. (B) Optimization of the washing conditions (shaking rate and NaCl concentration in the washing buffer). 1 ng of purified C-P4H was used as target. The error bars show the ± SD of three parallel experiments.

The influence of different parameters (hybridization time, salt concentration in the washing buffer and shaking rate) was studied to optimize the sensitivity, accuracy and reproducibility of the assay (Figure 4). The responses were presented as absolute signal minus background signal instead of signal to background ratio used earlier in order to visualize better the effect of the changed parameters. The highest fluorescent signal compared to the background level and the lowest standard deviation were obtained when the protein was incubated with each antibody for 1 h. Although a longer hybridization (more than 1 h) could positively effect on the signal, it was not tested, since it would noticeably increase the total assay time. The shaking rate during the hybridization step did not significantly influence the results (Figure 4A), and 700 rpm shaking rate was selected because it prevents sedimentation of the beads. An elevated concentration of NaCl in the washing buffer was expected to increase washing efficiency, but it did not have a significant effect on the signal (Figure 4B). It was obvious that plates need to be shaken during the washing phases between the hybridization steps. Without shaking the beads clumped and the washing was not efficient enough causing increased variation between the replicates (Figure 4B).

### Optimization of the antibody concentrations

The concentrations of the antibodies used in the sandwich ELISA were optimized using an experimental design approach.

The full factorial design of response surface methodology (RSM) was chosen to explore the optimal level of the three antibodies (Table 1). FLU signal/background ratio was measured with the sandwich ELISA for 1, 20 and 50 ng of C-P4H and used as the response (Additional file 1, table S1). The data was fitted with multiple regression to a second-order polynomial model equation using MODDE 8 software.

**Table 1:** Coded values for antibody concentrations

Coded values	Antibody dilutions		
	mab-α	pab-β	GAR-AP
-1	1/1000	1/1000	1/10000
0	1/500	1/500	1/5000
1	1/250	1/250	1/2500

The analysis of variance (ANOVA) was applied to test the fit of the model equation significance. The importance of each variable and their interactions were evaluated using *P*-values. *P*-values less than 0.05 indicate that model terms are significant (5 % significance level). In the case of measuring 1 ng of C-P4H, coded values of each antibody dilution, interaction between mab-α and GAR-AP dilutions and pab-β dilution quadratic coded value are significant model terms (Additional file 2, table S2). The model statistical significance was checked by F-tests (Additional file 3, table S3). The first F-test, comparing modellable and unmodellable variance, was satisfied since its *P*-value was < 0.05. The second, *lack of fit*, test, comparing the model and the replicate errors with each other, was also satisfied since its *P*-value was > 0.05. In addition, the model was evaluated using other statistical parameters showing that the model is good (Additional file 3, table S3).

The models created on the basis of signal/background ratio for 20 and 50 ng of C-P4H had similar parameters with the presented model for 1 ng of C-P4H (data not shown).

Using the MODDE 8 software optimizer tool the best antibodies concentrations were chosen (Table 2). The model indicates that the mab-α antibody concentration is the most important parameter and it has to be set at the highest level. Most probably, it is possible to get a better response using an even less diluted antibody, but it has not been done because the mab-α antibody is the most expensive reagent of the assay. To reduce the method significantly, the fourth parameter, mab-α antibody cost, was included in the optimizer. After this correction, the following antibodies dilutions were selected: 1/500 for mab-α, 1/700 for pab-β and 1/3000 for GAR-AP.

**Table 2:** Optimal antibody concentrations

Amount of C-P4H, ng	FLU signal/background	Dilutions of antibody		
		mab-α	pab-β	GAR-AP
1	13.37	1/250	1/770	1/2500
20	75.78	1/250	1/710	1/2500
50	88.77	1/250	1/830	1/3300

**Figure 5:**
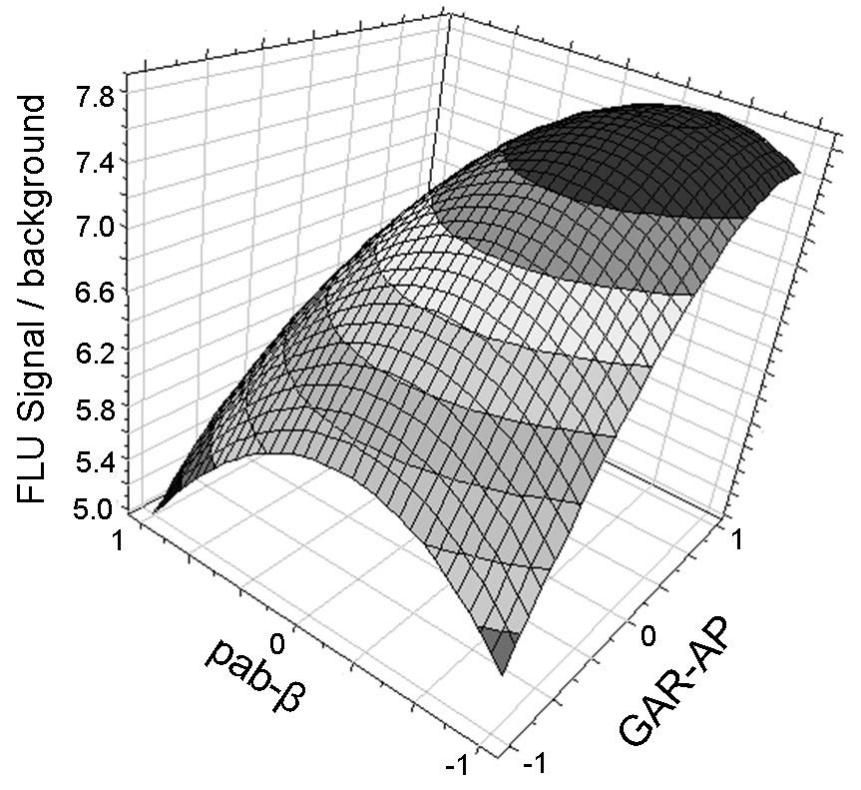
**The response surface plot showing the effects of pab-β and GAR-AP concentrations**. The FLU signal to background ratio was used as a response; mab-α concentration was set at the level of 0 (dilution 1/500). The antibody dilutions are presented in their coded values (Table 1).

**Figure 6:**
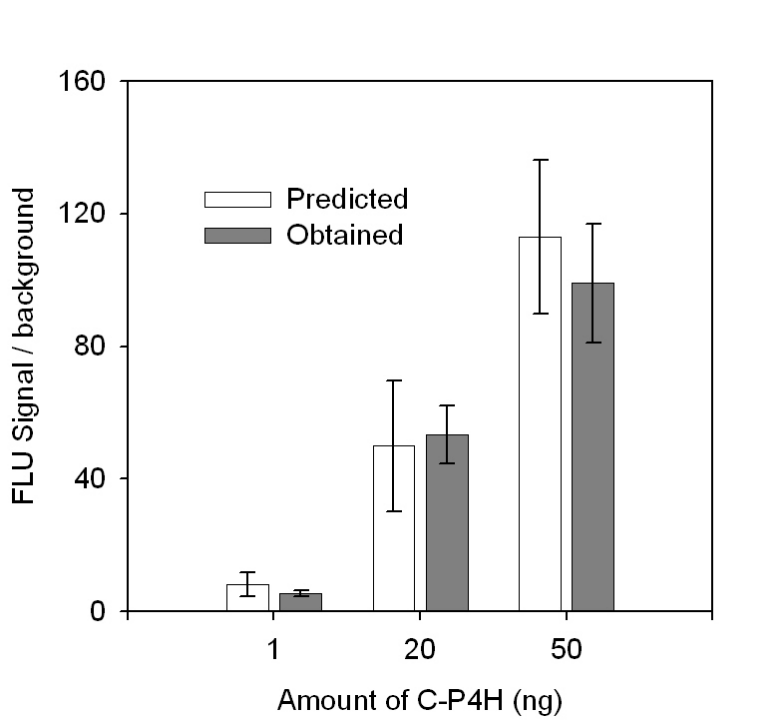
**Comparison of the responses predicted by the model and obtained in the experiment**. The purified C-P4H was used as a target. The antibody dilutions: mab-α 1/500, pab-β 1/700, GAR-AP 1/3000. The error bars show the ± SD of three parallel experiments.

The response surface plot shown in figure 5 illustrates the influence of the parameters pab-β and GAR-AP on the signal to background ratio. This plot was obtained for parameter mab-α which was fixed at the dilution 1/500. Finally the test was performed using the selected antibodies dilutions, and the results were shown to be close to predicted values of signal to background ratio (Figure 6).

### Sensitivity and range

**Figure 7:**
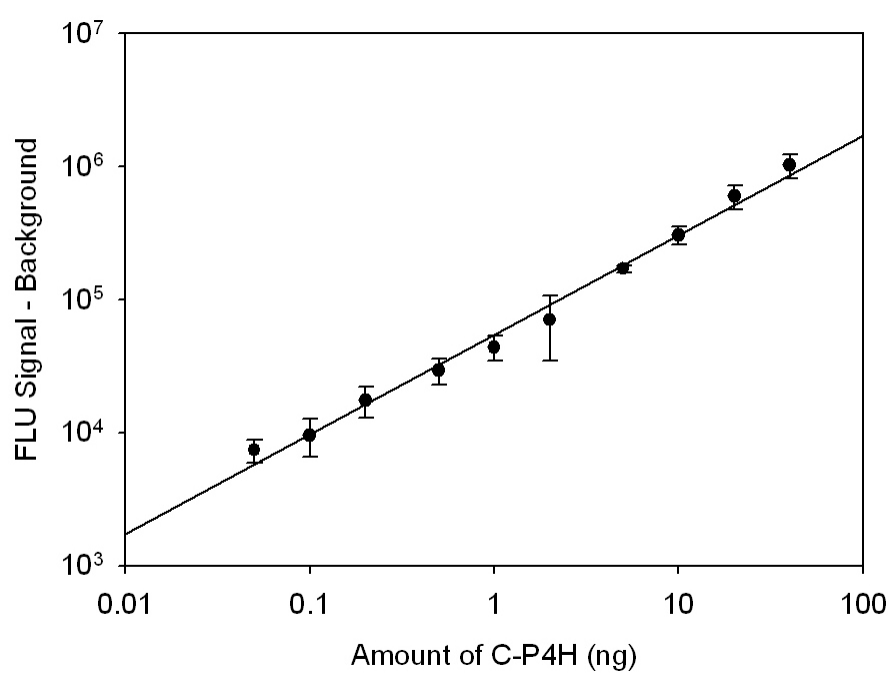
**Standard curve of the bead-based sandwich ELISA for C-P4H**. 0 - 40 ng of purified C-P4H were used as a target. The error bars show the ± SD of three parallel experiments

The assay sensitivity level and the range were determined using 0 - 40 ng of purified C-P4H tetramer (Figure 7). To simulate the natural background, 1 μg of *E. coli *whole cell extract was added to each sample. The detection limit was estimated to be 0.1 ng of C-P4H. This number corresponds to signal which is above the average of the background signal for three times of the blank signal standard deviation. The dependence observed between the C-P4H amount and the fluorescent signal can be used for C-P4H concentration estimation in real samples. The response was presented as the absolute signal minus the background signal in order to be able to estimate protein amount in samples more precisely.

### Analysis of the recombinant C-P4H expression in *Escherichia coli*

**Figure 8:**
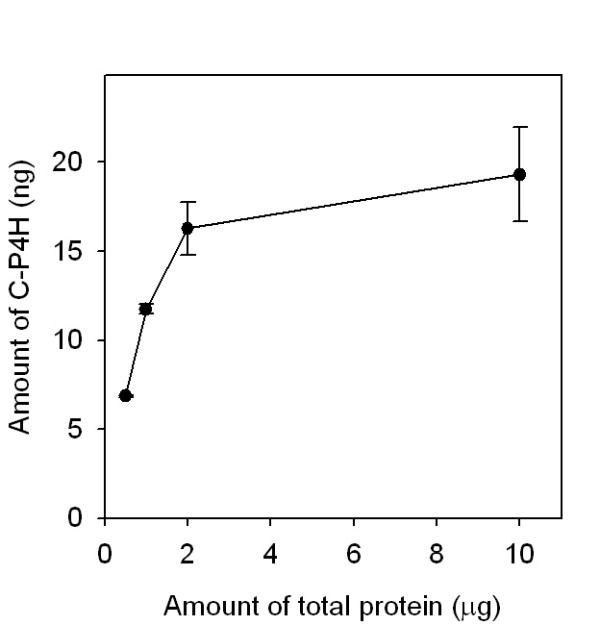
**The effect of cell lysate on the signal of the sandwich ELISA**. The amount of the C-P4H was measured in 0.5 - 10 μg of extract of *E. coli *cells expressing C-P4H. The error bars show the ± SD of three parallel experiments.

The effect of biological material on the signal of the sandwich ELISA was studied by measuring the amount of C-P4H in different dilutions of cell extracts of *E. coli *cells expressing C-P4H (0.5 - 10 μg of total proteins). The cells expressing C-P4H were cultivated and disrupted as explained in the Methods, diluted with the main buffer and used as a target for the sandwich ELISA in amount of 10 μL, corresponded to 0.5 - 10 μg of total proteins. The inhibition effect was observed with increasing of the total proteins concentration in the samples (Figure 8). To minimize this effect, the amount of total cell proteins analyzed with the assay should not exceed 1 μg.

**Figure 9:**
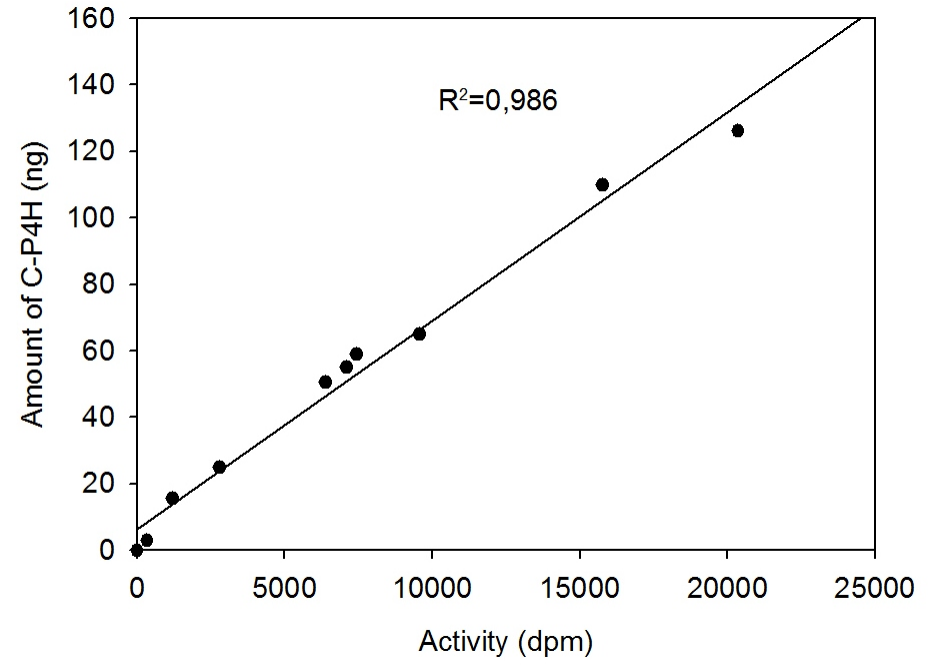
**Correlation between the C-P4H activity detected with the radioactive assay and the C-P4H amount measured with the bead-based sandwich ELISA**. The plotted amounts of C-P4H and activity units were presented per 10 μg of total *E. coli *protein.

The results obtained with the sandwich ELISA for C-P4H were compared with an activity assay based on the formation of 4-hydroxy[^14^C]proline in a [^14^C]proline-labeled substrate consisting of nonhydroxylated procollagen polypeptide chains [17]. The activity of 10 crude samples of C-P4H produced in *E. coli *was measured. The same samples were analyzed with the sandwich ELISA for C-P4H and the results were compared as shown in figure 9. A linear function and correlation coefficient of R^2 ^= 0.986 were observed demonstrating high correlation between the two methods. The observed correlation is valid only for the performed experiments, since the correlation between specific activity and amount of enzyme maybe different under different conditions such as expression levels, cell lysis or production conditions.

### Optimization of a fed-batch fermentation process for C-P4H production in *Escherichia coli*

The developed method was used in optimization of a fed-batch fermentation process of recombinant C-P4H production by *E. coli*. The experiment aimed at evaluating the effect of a lowered oxygen transfer rate after the first induction (IPTG) and the influence of the cell density at the first induction time on the produced enzyme amount. It was proposed that a lower oxygen transfer rate might exhibit a positive influence on the recombinant C-P4H expression by *E. coli*. In contrast to shake flask cultures where anhydrotetracyclin (aTc) caused a long-term α subunit induction, aTc induction was only transient in bioreactor cultures with strong aeration, possibly by an oxidation and thereby inactivation of the inducer aTc [20]. Furthermore, the production host for C-P4H was the *E. coli *Origami, a double mutant (*trxB gor*), which also might benefit from lower culture oxygenation. This mutant is heavily disturbed in the cytoplasmic redox system and only grows reasonably if combined with a suppressor mutation in the *ahpC *gene. However these strains show a high constitutive expression of the transcriptional regulator of the oxidative stress response, OxyR [21]. Therefore, based on the comparison of earlier results for cell growth and C-P4H overexpression in shake flasks and in bioreactors with a very much higher oxygen transfer rate, we have earlier suggested that a low oxygenation level may decrease this oxidative stress [16].

In order to avoid laborious cultivations in bioreactor the fed-batch conditions were performed in shake flasks using a glucose auto-delivery system (EnBase^®^) which simulates the conditions of a glucose limited fed-batch with constant glucose supply rate [22].

The high value for the shaking rate was chosen to be 220 rpm as in the earlier experiments. The low value was set to 100 rpm after the first induction in order to create a significant change in comparison to the high value without causing a shift to anaerobic metabolism or a shock due to extreme oxygen limitation. The shaking rate before the inductions was kept at 220 rpm in the each experiment.

The induction with IPTG was performed either at an OD_600 _of approximately 3 or at OD_600 _= 10. The cultivation conditions for the experiments are summarized in table 3. Each combination of parameter values was repeated once.

**Table 3:** Optimization of C-P4H production: summary of cultivation conditions

shaking rate [rpm]	1^st ^induction at OD_600 _=
220 (high level)	3 (low level)
220 (high level)	10 (high level)
220, 100 after 1^st ^induction (low level)	3 (low level)
220, 100 after 1^st ^induction (low level)	10 (high level)

**Figure 10:**
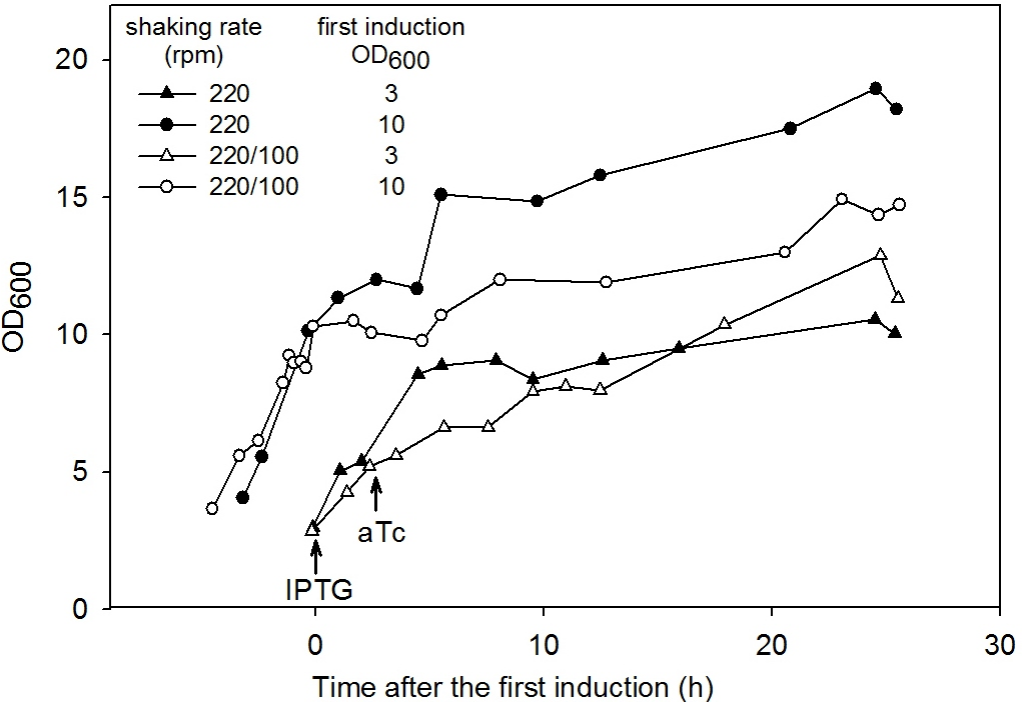
**The growth of *E. coli *produced recombinant C-P4H under different cultivation conditions**. The cells were cultivated with using EnBase^® ^technology, varying shaking rate and induction time. The arrows indicate the inductions of the PDI/β and α subunits expression with IPTG and aTc, respectively

The cell growth of the cultures is shown on figure 10. The higher oxygen transfer led to a prolongation of the exponential phase which was followed by a temporary growth decrease. Induction at OD_600 _= 10 led to final cell densities that were on an average 40% higher than the values achieved by earlier induction.

**Figure 11:**
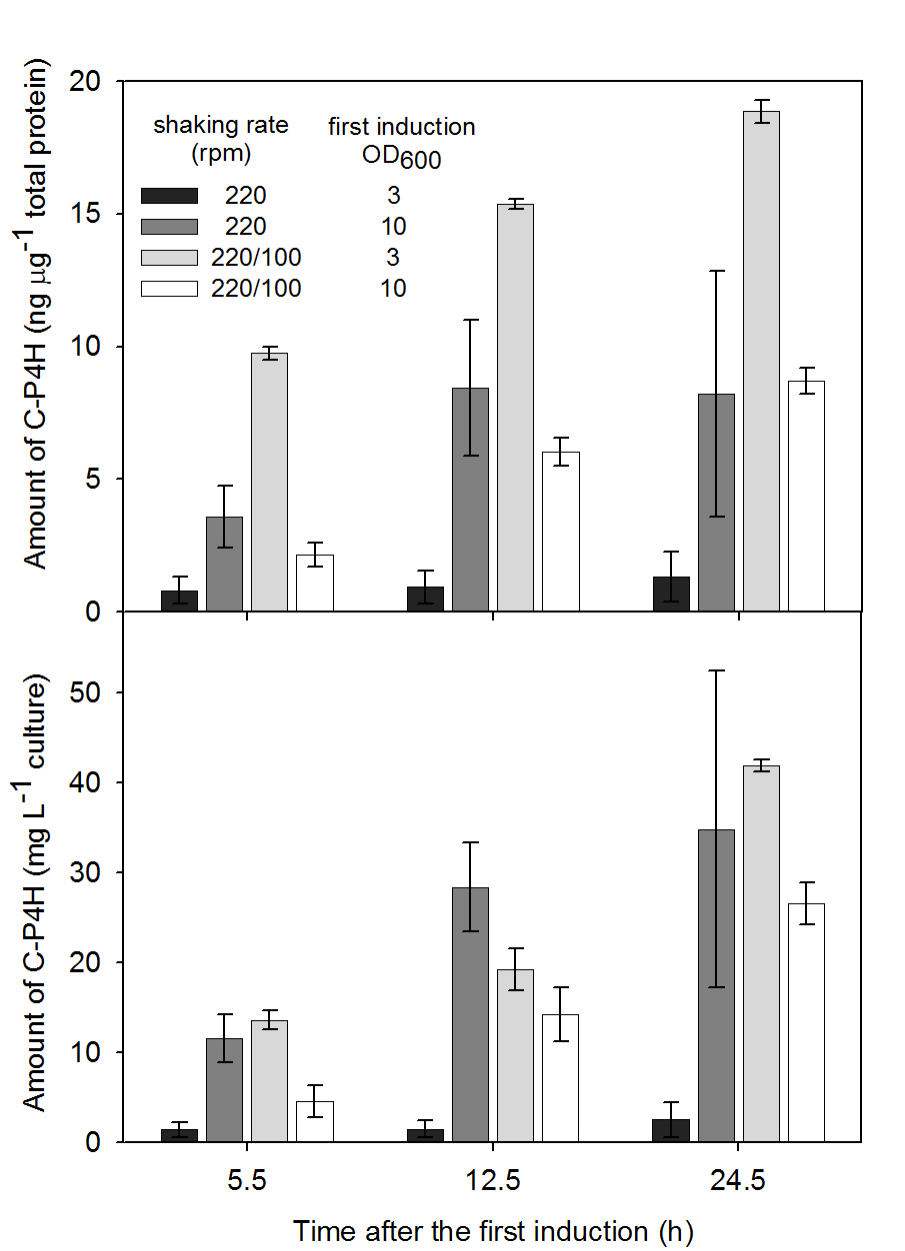
**C-P4H production by *E. coli *cultivated with using EnBase^® ^technology, varying shaking rate and induction time**. Experiments with identical conditions are presented as average values with error bars indicating 2 effective values from the analysis. The upper part: the C-P4H amount (ng) measured in 1 μg of total cell protein. The lower part: the C-P4H concentration (mg per one litre of the cultivation media).

The C-P4H production was monitored at 5.5, 12.5 and 24.5 h after the first induction using the developed ELISA (Figure 11). The experiments with high shaking rate and early induction showed the lowest product yield, slightly increasing over time (black bars). Induction with IPTG at an OD_600 _of 10 instead of 3 increased the protein production considerably (dark grey bars). With this combination of parameters (high shaking rate and late induction), the highest values after 12.5 h in relation to the culture volume were achieved (lower part of figure 11). The cultivations with reduced shaking rate after carrying out the first induction at OD_600 _= 3 resulted in the highest overall product yield relating to the total cell protein amount (light grey bars, upper part of figure 11). When the first induction was performed at high level (OD_600 _= 10) and the shaking rate lowered to 100 rpm subsequently, the second lowest product yield in comparison to the other experiments was obtained (white bars). There was a linear increase of C-P4H expression per total protein over the cultivation time.

The significance of the factors (shaking rate and induction time) was evaluated statistically using MODDE 8 software (Table 4). A positive factor effect means that the changing of the factor value leads to significant response changes. It is obvious that the factors were dependent of each other since there was always an interaction between the variables. Furthermore, the continuous shaking rate of 220 rpm had a negative influence on the protein production per cell at all sampling times. The effect induction time was not evident in the most of cases.

**Table 4:** Effect of shaking rate and OD_600 _at the first induction on C-P4H production

	C-P4H (ng μg^-1 ^total protein)		
	5.5	12.5	24.5
Shaking rate	-	-	-
OD_600 _at the fist induction	-	(-)	(-)
interaction	yes	yes	yes
	C-P4H (mg L^-1^)		
	5.5	12.5	24.5

Shaking rate	(-)	(-)	(-)
OD_600 _at the fist induction	(-)	+	(+)
interaction	yes	yes	(yes)

- negative effect of high level parameter

+ positive effect of high level parameter

() effect is not significant, tendency is shown in parentheses

yes interaction exists

## Discussion

A method for quantitative C-P4H holoenzyme detection in crude cell extracts was developed for monitoring of recombinant C-P4H production. C-P4H coexpression is required for production of recombinant collagens at that the enzyme's activity is a critical parameter [8,9,11-14]. Therefore C-P4H concentration monitoring of becomes important for the quality control in recombinant collagen processes. The α subunit of the enzyme has a high aggregation tendency, which may cause low amounts of functional enzyme and consequently low quality of the produced collagen, i.e. temperature instability. The standard test for C-P4H activity monitoring is measuring hydroxylation of peptides with radioactive substrate.

When used with other antibodies the here developed method can be principally adapted for the quantification of other protein complexes such as multimeric enzymes, receptors, ion channels etc., in crude or impure samples. The limitations of the required antibodies availability can be evaded in future due to the resource gained within the human protein atlas project [23].

The developed assay is a sandwich ELISA based on C-P4H tetramer hybridization with a biotin labeled capture antibody against its α subunit and a detection antibody against the PDI/β subunit. The antibodies specificity against the different C-P4H subunits ensures entire holoenzyme detection. During the assay the C-P4H is captured through its α subunit and all other molecules including the free PDI/β subunit are washed away. As a rule, tested samples contain abundance of PDI since it is initially transcribed during the protein expression [14] and it is also presented in excess amounts in relation to the α subunit *in vivo*.

An experimental design approach applied for the optimal antibodies concentration selection is presented in this study. A full factorial design was chosen because of the possible interactions between the parameters. The model obtained was tested by ANOVA and it proved to be statistically significant. The model indicated that the capture antibody concentration is the most effective parameter, which had to be set at its maximum level. This means that better results could be observed using the less diluted capture antibody, but taking into consideration its high cost 1/500 mab-α dilution was used. For the other antibodies the optimal levels were selected by the model and illustrated as a peak on the response surface plot (Figure 5). The signal/background values predicted by the model correlated well with the values which were obtained in a real assay (Figure 6).

The sandwich ELISA sensitivity was determined to be approximately 0.1 ng of C-P4H and the assay ranged between 0 and 40 ng of the pure protein.

A high amount of *E. coli *cell proteins had a negative effect on the signals obtained with the sandwich ELISA. This could be caused by antibodies blocking with cell proteins or aggregates of the C-P4H α and β subunits. To minimize this effect the amount of the total cell protein used for one measurement should not exceed 1 μg.

The developed sandwich ELISA was compared to the current standard, a radioactive assay which was mainly used for monitoring of the recombinant C-P4H assembly and activity during its production in different expression systems [17]. The measured concentrations of the C-P4H produced by *E. coli *cells correlated well with the amount of the enzyme activity (Figure 9). Therefore, as the sandwich ELISA is a quantitative and simple method for C-P4H measuring in crude samples, it is very suitable for high-throughput analyses instead of the technically demanding and time consuming radioactive assay.

The sandwich ELISA was applied for C-P4H level measuring in experiments aimed at optimizing of the C-P4H production by *E. coli *cells. The highest specific yield of active recombinant C-P4H up to now in cultivation with the Origami strain has been reported by Neubauer et al. [16]. Using the EnBase^® ^glucose auto-delivery system [22] we were able to obtain fed-batch conditions in shake flasks and succeeded to produce a high level of C-P4H tetramer in the shake flask scale (maximum product yield per culture volume was 42.5 mg L^-1^). It was shown that the oxygen transfer rate, the time for the first inducer (IPTG) addition and the interaction between these factors have a significant influence on the C-P4H production. In this study the maximum product yield was obtained under the following conditions: when the first induction (with IPTG) was performed at an OD_600 _of 3 and the oxygen transfer rate was reduced after the induction. These results may be treated as indirect proof for the supposition that the inducer aTc can be inactivated by a good oxygenation of the cultivation medium. Although not in the focus of the article, we propose that cultivations performed with aTc should be carried out under oxygen limitation.

A commercially available immunoassay for the serum C-P4H detection utilizes only antibodies specific for the PDI/β subunit and thus detects also free PDI/β-subunits [19]. As PDI is a common and copious enzyme in the endoplasmic reticulum, this method fails to analyze the C-P4H amount or activity. In the method developed here only the C-P4H tetramers are measured due to the fact that antibodies against both subunits are used for the protein capturing and detection. The described sandwich ELISA has a potential to be a tool for the assembled C-P4H quantification in blood at fibrotic diseases detection.

## Conclusions

The method developed in this study is relevant to quantitative collagen prolyl-4-hydroxylase detection for monitoring the recombinant C-P4H production and C-P4H coexpression during recombinant collagens production. The specificity of the antibodies used in the assay against the different collagen prolyl-4-hydroxylase subunits ensures detection of the holoenzyme only, but not its separate subunits. Using an experimental design approach the antibody concentrations in the ELISA were optimized. The method was successfully applied for monitoring the recombinant C-P4H amount in crude cell extracts of *E. coli *during the optimization of C-P4H production. This study demonstrates the sandwich ELISA applicability for quantifying of proteins consisting of different subunits in crude samples. The method is potential for recombinant multimeric proteins production monitoring as well as complex proteins detection in clinical and food diagnostics.

## Methods

### Strain and cultivation conditions

For production of recombinant C-P4H the strain *E. coli *Origami™ (*Δ*(*ara-leu*)*7697 araD139 ΔlacX74 aphC galE galK rpsL ΔphoA PvuII phoR F*'[*lac*^+^(*lacI^q^*)*pr*o] *gor522*::Tn*10 *(Tc^R^) *trxB*::kan, Novagen) carrying a vector for cytoplasmic expression of recombinant C-P4H (pP4Hcyt) was used [16]. Shake flask cultivations were performed in a modified LB medium containing 20 g L^-1 ^tryptone, 10 g L^-1 ^yeast extract, 5 g L^-1 ^NaCl and 100 mg L^-1 ^ampicillin. Exponentially grown 10 mL precultures inoculated with glycerol stocks carried out in 100 mL Erlenmeyer flasks at 37°C and 220 rpm on a rotary shaker served as an inoculum for the main cultures, which were performed in 3-baffled 1 L Erlenmeyer flasks with a liquid volume of 100 mL. The cultivations were carried out on a rotary shaker at 220 rpm and 25°C and were set to 20°C after the second induction [14]. When the cell density (OD_600_) reached 0.2, expression of the PDI/β subunit was induced with 50 μM isopropyl-β-d-thiogalactoside (IPTG), and when it reached 0.6, expression of the α subunit was induced with 200 μg L^-1 ^anhydrotetracycline hydrochloride (aTc, IBA GmbH).

The cultivations aimed at C-P4H production optimization were performed using gel-based EnBase^® ^technology (Biosilta Oy, Oulu, Finland). EnBase^® ^technology supplies the cell culture continuously with glucose and thus allows glucose limited fed-batch cultivation in a closed system [22,24]. Pre-cultures were prepared as described earlier. The cultivations were carried out in 3-baffled 1 L Erlenmeyer flasks with a liquid volume of 100 mL. The glucose-free medium containing 2.0 g L^-1 ^Na_2_SO_4_, 2.5 g L^-1 ^(NH_4_)_2_SO_4_, 0.50 g L^-1 ^NH_4_Cl, 14.60 g K_2_HPO_4_, 3.60 g L^-1 ^NaH_2_PO_4 _· 2H_2_O, 1.00 g L^-1 ^(NH_4_)_2_-H-citrate, 3 mM MgSO_4_, 0.1 g L^-1 ^thiamine hydrochloride, 2 ml L^-1 ^trace element solution (containing: 0.50 g L^-1 ^CaCl_2 _· 2H_2_O, 0.18 g L^-1 ^ZnSO_4 _7H_2_O, 0.10 g L^-1 ^MnSO_4 _· H_2_O, 20.1 g L^-1 ^Na_2_-EDTA, 16.70 g L^-1 ^FeCl_3 _· 6H_2_O, 0.16 g L^-1 ^CuSO_4 _· 5H_2_O and 0.18 g L^-1 ^CoCl_2 _· 6H_2_O) and 100 mg L^-1 ^ampicillin was used. The expression of the PDI/β subunit was induced with 50 μM IPTG with OD_600 _reached 3 or 10, and after 2 h the expression of the α subunit was induced with 200 μg L^-1 ^aTc. The cultivation temperature of 25°C was lowered to 20°C after the 2^nd ^induction. The cultivations were carried out on a rotary shaker at 220 rpm. For some cultivations the shaking rate was lowered to 100 rpm after the 1^st ^induction.

### Analysis of recombinant C-P4H

Cells were harvested by centrifugation at 20,000 × *g *for 5 min at 4°C at different time points before and after the inductions. The size of sample was calculated so that it would contain a quantity cells corresponding to 40 mL of OD_600 _= 1. The pellets were suspended in 2 mL of the lysis buffer (0.1 M NaCl, 0.1 M glycine, 10 μM dithiothreitol, 10 mM Tris-HCl, pH 7.8) containing complete EDTA-free protease inhibitor cocktail (Roche). The suspension was sonicated for 4 × 1 min at 1 min intervals on ice (Sonifier cell disruptor B-30, Branson, 3 mm diameter of sonotrode, output control 5) and centrifuged at 15,000 × *g *for 15 min at 4°C. Soluble cytoplasmic fractions were collected. Recombinant C-P4H was purified by a procedure consisting of a poly (L-proline) affinity chromatography and anion exchange chromatography on a HiTrap Q sepharose column (Amersham Biosciences; [25]).

C-P4H activity in the *E. coli *fractions was analysed by a method based on the formation of 4-hydroxy[^14^C]proline in a [^14^C]proline-labelled substrate consisting of nonhydroxylated procollagen polypeptide chains [17]. Protein concentrations were determined with the RC DC Protein Assay Kit (Bio-Rad Laboratories).

### Antibodies

An Anti-Human Prolyl-4-Hydroxylase (α) Mouse Monoclonal Antibody, clone 9-47H10 (MP Biomedicals, LLC) was used as the capture antibody (mab-α) for sandwich ELISA. Rabbit Anti-Human PDI Polyclonal Antibody, Abcam (pab-β A), Anti-Protein Disulfide Isomerase Rabbit pAb, Calbiochem, Germany (pab-β B), PDI (H-160) polyclonal rabbit antibody, Santa Cruz Biotechnology, Inc. (pab-β C) and Rabbit Anti-PDI Polyclonal Antibody, Stressgen Bioreagents (pab-β D) were tested as detection antibodies. Alkaline Phosphatase-conjugated AffiniPure Goat Anti-Rabbit IgG (H+L) (GAR-AP) and Phosphatase-conjugated AffiniPure Goat Anti-Mouse IgG (H+L) (GAM-AP), Jackson ImmunoResearch Laboratories, Inc. were used as the secondary detection antibody.

The dilutions of the antibodies were done with the main buffer (1 × PBS (1.8 mM KH_2_PO_4_, 10 mM Na_2_HPO_4_, 140 mM NaCl, 2.7 KCl, pH 7.4) with 1% BSA).

The capture antibody (mab-α) was biotinylated with EZ-Link™ Sulfo-NHS-LC-Biotin, PIERCE, USA. 13.3 μl of the antibody was incubated with 1 ml of 10 mM biotin reagent, diluted in the PBS buffer, at 25°C for 1 h. The solution was dialysed using Slide-A-Lyser^® ^Mini Dialysis Units (10,000 MWCO), PIERCE, USA, in PBS buffer during 1 h.

### Sandwich ELISA (optimized protocol)

The analysis was carried out in U-shaped transparent 96-well microtiter plates (Greiner Bio-One, Frickenhausen, Germany). The wells were incubated with 120 μL of the main buffer (1 × PBS (1.8 mM KH_2_PO_4_, 10 mM Na_2_HPO_4_, 140 mM NaCl, 2.7 KCl, pH 7.4) with 1% BSA) for 30 min at 25°C and 700 rpm in a Thermomixer comfort (Eppendorf). After the incubation, the buffer was discarded and the plate was allowed to dry. The wells were incubated (30 min, 25°C, 700 rpm) with 85 μL of biotin-labeled capture antibody (mab-α) diluted 1:500 and 15 μL of streptavidin coated paramagnetic beads [26,27], previously washed 3 times in the main buffer. Before adding 100 μL of the purified protein or samples, the wells were washed once with a washing solution (1 × PBS containing 1 % BSA and 0.05 % Tween20). The washing solution was incubated in the plate for 1 min (25°C, 750 rpm) and then removed while the magnetic beads were retained in the wells by a magnet. After incubation of the samples (1 h, 25°C, 700 rpm), the wells were washed twice, 100 μL per well of the first detection antibody (pab-β) diluted 1:700 was added and incubated in the plate (1 h, 25°C, 700 rpm). 100 μL of the secondary detection antibody (GAR-AP) diluted 1:3000 was added after 3 more washing steps and incubated (1 h, 25°C, 700 rpm). After the incubation, the wells were washed 3 times and the liquid from the last washing step was transferred into a new plate together with the beads. The wells of the new plate were washed once more.

In order to obtain a fluorescence signal, 100 μL of the AttoPhos^® ^(BBTP) substrate was added into each well and the enzymatic reaction was allowed to take place while incubating the plate for 20 min (37°C, 700 rpm, protected from light). The measurement was performed with 90 μL of the liquid from each well in a black microtiter plate (OptiPlate™-96F, PerkinElmer^TN ^Life Sciences, USA) using a Wallac Victor^2 ^1420 Multilabel counter (PerkinElmer^TN ^Life Sciences, USA) at an excitation wavelength of 430/450 nm and an emission wavelength of 560 nm.

### Experimental design and statistical analysis

A 3^3 ^full factorial design was used to optimize the antibodies dilutions for C-P4H sandwich ELISA. The center points of the parameters corresponded to previously used concentrations, and double diluted and double concentrated solutions of the antibodies were tested. These concentrations were logarithmically transformed and coded as 0, -1 and 1, respectively for statistical calculations (Table 1). The levels of the variables and the experimental design are shown in additional file 1, table S1. In this study, the experiment design contains 60 trials including 3 center points and 1 replicate of each trial. The ratio of fluorescence signal to background obtained for 1, 20 and 50 ng of C-P4H was selected as a response value. The second-order polynomial model was created and analyzed using MODDE 8 software (Umetrics, Sweden).

## Competing interests

The authors declare that they have no competing interests.

## Authors' contributions

EO designed the experiments and carried them out for the method development, optimization and testing. AN participated in the method development and performed the experiments for C-P4H production and its detection with the radioactive assay. KK performed experiments for optimization of C-P4H production. JM contributed with ideas and discussions in the design of the experiments and the writing of the manuscript. PN participated in the design and supervision of the work. All authors read and approved the final manuscript.

## Supplementary Material

Additional file 1 **3^3 ^full factorial design for the antibody concentration optimization**. Click here for file

Additional file 2 **ANOVA for the antibody concentration model**. A parameter is significant if its *P*-value is lower 0.05. *X_1_*, *X_2 _*and *X_3 _*are coded dilutions of mab-α, pab-β and GAR-AP antibody, respectively. Click here for file

Additional file 3 **The evaluation of the antibody concentration model**. Click here for file
