# Supplementary material for: Sandwich ELISA for quantitative detection of human collagen prolyl 4-hydroxylase
Source: Microb Cell Fact. 2010 Jun 17;9:48. doi: 10.1186/1475-2859-9-48 (PMC2895579; doi:10.1186/1475-2859-9-48)
Supplement: Additional file 1 — 33 full factorial design for the antibody concentration optimization. [file 1475-2859-9-48-S1.PDF]

Table S1 - 3^3 full factorial design for the antibody concentration optimization

Coded values of antibody dilutions			FLU Signal / background					
			1 ng C-P4H		20 ng C-P4H		50 ng C-P4H	
mab-α	pab-β	GAR-AP	Tr. 1	Tr. 2	Tr. 1	Tr. 2	Tr. 1	Tr. 2
-1	-1	-1	4.5	1.9	25.9	11.5	61.7	16.0
-1	-1	0	3.9	2.4	31.6	9.6	44.4	9.5
-1	-1	1	3.2	3.3	20.8	18.6	59.3	41.6
-1	0	-1	3.4	3.2	23.5	14.8	45.1	24.0
-1	0	0	3.4	2.9	28.9	19.1	43.7	35.0
-1	0	1	5.2	3.6	31.3	26.4	52.0	30.0
-1	1	-1	3.3	2.0	30.5	18.2	34.1	25.5
-1	1	0	4.1	2.8	32.0	22.9	50.9	24.8
-1	1	1	3.1	2.9	13.2	18.8	26.9	30.5
0	-1	-1	5.4	7.0	24.0	46.0	40.2	65.8
0	-1	0	9.4	7.7	58.3	50.3	100.8	75.4
0	-1	1	6.9	8.6	36.3	50.6	47.1	72.4
0	0	-1	5.4	6.7	31.4	46.1	52.7	61.9
0	0	0	7.8	6.7	36.2	49.4	47.8	74.7
0	0	1	8.4	6.5	53.8	46.2	46.9	68.4
0	0	0	6.7	6.1	48.0	44.6	75.7	79.9
0	0	0	10.0	4.7	63.7	33.8	114.1	53.4
0	0	0	9.1	7.1	55.9	44.5	64.2	66.0
0	1	-1	4.7	4.7	35.8	40.6	49.3	49.5
0	1	0	6.1	5.8	37.8	32.6	62.1	55.4
0	1	1	4.1	5.1	24.1	32.9	36.1	49.3

1	-1	-1	8.4	7.1	65.2	46.1	88.4	25.3
1	-1	0	13.5	12.2	77.6	63.7	91.4	88.2
1	-1	1	11.8	13.7	73.3	64.0	94.0	82.2
1	0	-1	10.4	11.2	65.1	63.5	76.0	88.4
1	0	0	11.4	15.5	60.5	84.6	76.6	81.2
1	0	1	15.9	10.0	90.7	69.7	96.1	78.2
1	1	-1	10.6	8.5	63.5	55.7	87.2	59.8
1	1	0	11.2	7.7	67.8	49.9	54.6	74.1
1	1	1	14.1	9.0	76.2	51.4	80.8	58.6
