# Supplementary material for: Sandwich ELISA for quantitative detection of human collagen prolyl 4-hydroxylase
Source: Microb Cell Fact. 2010 Jun 17;9:48. doi: 10.1186/1475-2859-9-48 (PMC2895579; doi:10.1186/1475-2859-9-48)
Supplement: Additional file 2 — ANOVA for the antibody concentration model. A parameter is significant if its P-value is lower 0.05. X1, X2 and X3 are coded dilutions of mab-α, pab-β and GAR-AP antibody, respectively. [file 1475-2859-9-48-S2.PDF]

**Table S2 - ANOVA for the antibody concentration model**

| Term | Effect | Coefficient | *P*-value | Significance |
|---|---|---|---|---|
| Constant | | 7.46889 | 1.76775e-023 | Yes |
| $X_1$ | 7.94348 | 3.97174 | 9.98059e-021 | Yes |
| $X_2$ | -1.16892 | -0.584462 | 0.0268867 | Yes |
| $X_3$ | 1.49567 | 0.747834 | 0.00527037 | Yes |
| $X_1^2$ | 1.62401 | 0.812004 | 0.0561372 | No |
| $X_2^2$ | -1.7841 | -0.892051 | 0.0365708 | Yes |
| $X_3^2$ | -1.29134 | -0.645671 | 0.12629 | No |
| $X_1X_2$ | -0.386096 | -0.193048 | 0.541357 | No |
| $X_1X_3$ | 1.28773 | 0.643867 | 0.0455131 | Yes |
| $X_2X_3$ | -0.735567 | -0.367783 | 0.246902 | No |

A parameter is significant if its *P*-value is lower 0.05. $X_1$, $X_2$ and $X_3$ are coded dilutions of mab-α, pab-β and GAR-AP antibody, respectively.
