# Supplementary material for: Sandwich ELISA for quantitative detection of human collagen prolyl 4-hydroxylase
Source: Microb Cell Fact. 2010 Jun 17;9:48. doi: 10.1186/1475-2859-9-48 (PMC2895579; doi:10.1186/1475-2859-9-48)
Supplement: Additional file 3 — The evaluation of the antibody concentration model. [file 1475-2859-9-48-S3.PDF]

Table S3 - The evaluation of the antibody concentration model

Parameter	Value	Critical value (to be a good model)	Meaning parameters
R^2	0.844	> 0.5	Explained variation, goodness of fit parameter
R^2_{adj}	0.816	> 0.5	Goodness of fit measure adjusted for degrees of freedom
Q^2	0.771	> 0.5	Predicted variation, goodness of prediction parameter
R^2 - Q^2	0.073	< 0.2 – 0.3	Appropriateness of a model
Model validity	0.929	> 0.25	Defines if this is the right type of a model
Reproducibility	0.797	> 0.5	Shows replicate error
F-test for mean-squares (*P*-value)	0.000	< 0.05	Compares modellable and unmodellable variance
Lack of fit test (*P*-value)	0.754	> 0.05	Compares model and replicate errors with each other
